# Mild hypercapnia improves brain tissue oxygen tension but not diffusion limitation in asphyxial cardiac arrest: an experimental study in pigs

**DOI:** 10.1186/s12871-020-01162-z

**Published:** 2020-09-29

**Authors:** Dawei Zhou, Zhimin Li, Shaolan Zhang, Lei Wu, Yiyuan Li, Guangzhi Shi, Jianxin Zhou

**Affiliations:** Department of Critical Care Medicine, Beijing Tiantan Hospital, Capital Medical University, No. 119 South Fourth Ring West Road, Beijing, 100070 China

**Keywords:** Cardiac arrest, Post cardiac arrest syndrome, Hypercapnia, Brain tissue oxygen tension, Diffusion limitation

## Abstract

**Background:**

We sought to evaluate the effect of mild hypercapnia on brain tissue oxygen tension (Pbto_2_) and diffusion limitation (impaired ability of oxygen extraction) in a porcine post asphyxial cardiac arrest model.

**Methods:**

In 16 Bama pigs, asphyxial cardiac arrest was induced by endotracheal tube clamping and remained untreated for another 4 min. After return of spontaneous circulation (ROSC), animals were randomly assigned to mild hypercapnia (end-tidal carbon dioxide (EtCO_2_): 45 ~ 50 mmHg) and normocapnia (EtCO_2_: 35 ~ 40 mmHg) groups for 12 h. Intracranial pressure (ICP), Pbto_2_, and brain tissue temperature were invasively measured by multimodality monitors. Blood gas analysis, neuron specific enolase (NSE), and S100β were tested at baseline, ROSC 1 h, 6 h, and 12 h. Generalized mixed model with a compound symmetry covariance matrix was used to compare the time-variables of the two groups.

**Results:**

Twelve (75%) pigs had ROSC and 11 pigs survived for the study period, with 6 pigs in mild hypercapnia group and 5 in the normocapnia group. The mean EtCO_2_ in the mild hypercapnia was significantly higher than normocapnia group (48 vs 38 mmHg, *p* <  0.001). Compared with normocapnia, mild hypercapnia group had higher Pbto_2_ (*p* <  0.001), slightly higher mean arterial pressure (*p* = 0.012) and ICP (*p* = 0.009). There were no differences in cerebral perfusion pressure (*p* = 0.106), gradient of partial pressure of jugular venous bulb oxygen (Pjvo_2_) and Pbto_2_ (*p* = 0.262), difference of partial pressure of jugular venous CO_2_ and arterial CO_2_ (*p* = 0.546), cardiac output (*p* = 0.712), NSE (*p* = 0.822), and S100β (*p* = 0.759) between the two groups.

**Conclusions:**

Short term mild hypercapnia post-resuscitation could improve Pbto_2_. However, no corresponding improvements in the gradient of Pjvo_2_ to Pbto_2_ and biomarkers of neurological recovery were observed in the porcine asphyxial cardiac arrest model.

## Background

Cardiac arrest (CA) is one of the major health problems worldwide, with significantly high mortality and morbidity [1–3]. Despite the return of spontaneous circulation (ROSC) after successful cardiopulmonary resuscitation (CPR), a majority of patients die prior to hospital discharge or have severe neurologic injury attributable to the development of post-cardiac arrest syndrome (PCAS) [4, 5]. PCAS, composed of four components: hypoxic brain injury, systemic ischemia reperfusion injury, myocardial dysfunction, and the underlying etiology of CA, is substantially complex to manage [6–8].

A number of specific interventions have been demonstrated to improve outcomes of PCAS, of which the management of the partial pressure of arterial carbon dioxide (Paco_2_) as a potential therapeutic target receives special attention [9–11]. Carbon dioxide is the major physiological regulator of cerebral blood flow (CBF), with higher Paco_2_ causing increased brain perfusion [12]. Previous studies found mild hypercapnia increased cerebral oxygenation assessed by near infrared spectroscopy (NIRS) and attenuated the release of serum neuron specific enolase (NSE) [9, 11, 13]. However, the results were inconsistent and the potential mechanisms are unclear [10, 14, 15]. In addition, NIRS may not accurately reflect the cerebral oxygenation [16].

The present study aimed to investigate the effect of mild hypercapnia on cerebral oxygenation assessed by invasive brain tissue oxygen tension (Pbto_2_) monitoring probe and diffusion limitation assessed by the gradient of partial pressure of jugular venous bulb oxygen (Pjvo_2_) and Pbto_2_ in a porcine asphyxial CA model. We hypothesized that mild hypercapnia might increase Pbto_2_ and improve diffusion limitation.

## Methods

The study was approved by the Institutional Review Board (No: 201803002) of Beijing Tiantan Hospital, Capital Medical University. All animals received humane care in compliance with the guideline for the care and use of experimental animals by the National Institute of Health [17].

### Animal preparation

Sixteen healthy, male Bama miniature pigs (weight 35 ~ 45 kg, mean 39.7 kg), purchased from Beijing Shichuang Minipig Breeding Base were studied. All animals were fasted overnight except for ad libitum access to water. Animals were placed on a heating pad in supine position. Premedication consisted of intramuscular 10 mg/kg ketamine. Anesthesia was induced by ear vein injection of propofol (1–2 mg/kg) and fentanyl (2 μg/kg). Continuous sedation and analgesia consisted of intravenous 4–8 mg/kg/h propofol and 1–5 μg/kg/h fentanyl. Lactated Ringer’s solution (5 ml/kg/h) was administrated as maintenance fluid.

Tracheotomy was performed with a 7 mm cuffed tube. Following securing the airway, animals were mechanically ventilated with a Servo-s ventilator (Maquet, Solna, Sweden) with a tidal volume of 10 ml/kg, respiratory rate (RR) of 16 breaths/min, and inspired oxygen fraction of 21%. The end-tidal carbon dioxide pressure (EtCO_2_) was monitored by an infrared capnometer (Mindray, BeneVision N19 with module of EtCO_2_) and EtCO_2_ of 35 and 40 mmHg was maintained by adjusting the parameters of the ventilator before establishing the asphyxial CA model.

A 7F central venous catheter was placed by surgical cut-down technique via right external jugular vein to the right atrium for fluid infusion, central venous pressure (CVP) measurement and bolus of 10 ml ice-cold saline injections for cardiac output (CO) measurement by transpulmonary thermodilution method. The position was ascertained by the shape of pressure wave in right atrium. A 5F catheter was retrogradely to the cranial direction inserted into the right internal jugular vein reaching an approximated tip location of the jugular bulb for blood collection. Furthermore, the tip location was confirmed by X-rays. Left femoral artery was inserted with a 5F arterial catheter with an integrated thermistor tip (PiCCO, Pulsion Medical Systems, Munich, Germany) for continuous mean arterial blood pressure (MAP) monitoring, arterial blood gas (ABG) sampling, extravascular lung water (EVLW), and CO measurement. Pressure related catheters were connected to transducers (Mindray, BeneVision N19 with module for PiCCO). A conventional electrocardiogram with five adhesive electrodes to the four shaved skin of the proximal limbs and the upper abdomen was continuously monitored.

After the procedures above, animals were turned to left lateral position. A midline incision was performed along the dorsal surface of the head to expose the underlying skull. One burr hole with diameter about 3 mm (mm) approximately 10 mm right and lateral of midline and 10 mm anterior to the coronal suture was drilled. The Pbto_2_ probe and temperature (Tbt) probe (Licox, INTEGRA, USA) were placed through the same opening hole. The distal tip of the probes were placed into the cortex at a depth of about 10 mm. The Pbto_2_ and Tbt monitor (Licox, INTEGRA, USA) were connected. Animals were then turned to right lateral position. Another burr hole was drilled at the symmetrical position on the left. An intraparenchymal ICP monitor probe (Codman Microsensor, Raynham, MA, USA) was placed through the hole. The distal tip of the probe was placed into the cortex at a depth of about 10 mm. The probe was connected to the ICP monitor (Codman, Raynham, MA, USA). The urinary catheter (22Fr Foley) was placed through bladder puncture for urine drainage.

### Experimental preparation

The animals were allowed to stabilize for 60 min after all the procedures above to achieve a stable resting level and then the baseline parameters were obtained.

### Establishment of the CA model

Intravenous rocuronium (0.5 mg/kg) was administrated for muscle relaxation. Asphyxia was induced by clamping the endotracheal tube. CA was confirmed when MAP less than 30 mmHg as previously described [18]. Anesthetic administration was ceased and the endotracheal tube was reopened after CA had been successfully induced.

### Cardiopulmonary resuscitation

After 4 min of untreated CA, CPR was initiated. Mechanical ventilation (volume-controlled ventilation, tidal volume 10 ml/kg, RR 10 breaths/min, Fio_2_ 100%) was resumed. In all animals, chest compressions were performed by the trained investigators at a frequency of 100 compressions/min with equal compression-relaxation duration and compression depth of one third of the anteroposterior diameter of the thorax according to resuscitation guideline [19].

Electrical defibrillation was performed with 150-J biphasic shocks using the defibrillator (Lifepak20e, Medtronic, USA) when ECG showed ventricular fibrillation (VF). If VF waves persisted, CPR was continued, followed by another electrical defibrillation (biphasic 200-J for subsequent attempts). After 2 min of CPR, epinephrine (0.02 mg/kg) was injected through the central venous catheter of external jugular vein. If required, additional doses of epinephrine were administered every 3 min until ROSC was achieved. Within 30 min, the above sequence continued until ROSC was achieved, which was defined as MAP more than 60 mmHg that was continuously sustained for at least 10 min. If ROSC was not achieved within 30 min, CPR was ceased and the animals were regarded as dead.

### Randomization and advanced life support

Twelve animals achieved ROSC. After ROSC, animals were randomized into normocapnia (EtCO_2_ 35 ~ 40 mmHg) and mild hypercapnia (EtCO_2_ 45 ~ 50 mmHg) ventilation using a sealed envelope indicating the animal assignment to either the mild hypercapnia group (*n* = 6) or the normocapnia group (*n* = 6). The investigators were blinded to the treatment allocation when assessing the outcome.

After ROSC, animals underwent intensive care for 12 h, and advanced life support was followed according to the guidelines [8]. Anesthesia, muscle relaxation, and Lactated Ringer’s solution were maintained. For ventilation, RR was adjusted to obtain an EtCO_2_ 35 ~ 40 mmHg in normocapnia group or EtCO_2_ 45 ~ 50 mmHg in the hypercapnia group when keeping tidal volume 10 ml/kg. For oxygenation, arterial oxygen saturation (Spo_2_) was titrated as 94% ~ 99%. Hemodynamic goal was set as MAP greater than 65 mmHg and norepinephrine was used when lower than the goal.

**Fig. 1:**
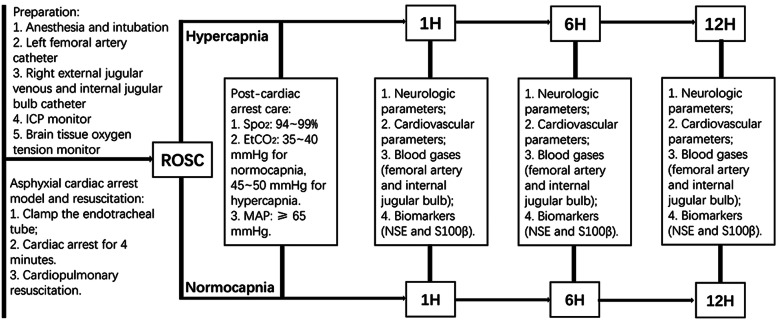
Experimental pipeline and procedures. ICP intracranial pressure, ROSC return of spontaneous circulation, H hour, Spo_2_ peripheral oxygen saturation, EtCO_2_ end-tidal carbon dioxide, MAP mean arterial pressure. NSE neuron-specific enolase, S100β, central nervous system specific protein

The experimental flow chart was summarized in Fig. 1. After intensive care for 12 h, all experimental animals were euthanized with a bolus of propofol 50 mg intravenous and then a bolus 20 ml of 20 mol/L potassium chloride.

### Measurements

Hemodynamics (MAP, CVP), electrocardiogram, EtCO_2_, and Spo_2_ data were continuously monitored using a patient monitoring system (Mindray, BeneVision N19) and were recorded every half an hour. CO and EVLW were measured at baseline, ROSC 1 h, 6 h, and 12 h with a bolus of 10 ml ice-cold saline injections for three times to obtain mean value, respectively.

Neurological parameters, such as ICP, Pbto_2_, and Tbt were continuously monitored and were recorded every half an hour until 12 h. Cerebral perfusion pressure (CPP) was calculated as MAP minus ICP (CPP = MAP - ICP).

Arterial and jugular venous bulb blood gas (GEM3000, Instrumentation Laboratory, USA) were analyzed with the temperature-corrected measurement at baseline, ROSC 1 h, 6 h, and 12 h. Pressure of arterial oxygen (Pao_2_), arterial carbon dioxide (Paco_2_), jugular venous bulb oxygen (Pjvo_2_), and jugular venous bulb carbon dioxide (Pjvco_2_), saturation of arterial oxygen (Sao_2_) and jugular venous bulb oxygen (Sjvo_2_), lactate of arterial and jugular venous bulb were recorded from the corresponding blood gas results (baseline, ROSC 1 h, 6 h, and 12 h). Gradient of Pjvo_2_ and Pbto_2_ was calculated as Pjvo_2_ minus Pbto_2_. Arteriojugular oxygen difference (AJDo_2_), difference of Pjvco_2_ to Paco_2_ (ΔPco_2_), lactate oxygen index (LOI) were calculated with the following equations:

$$ {\mathrm{AJDo}}_2=\left(\left[{\mathrm{Sao}}_2-{\mathrm{Sjvo}}_2\right]\times \left[\mathrm{Hemoglobin}\right]\times 1.34\right)+\left[\left({\mathrm{Pao}}_2-{\mathrm{Pjvo}}_2\right)\times 0.003\right]; $$

$$ \varDelta {\mathrm{Pco}}_2={\mathrm{Pjvco}}_2-{\mathrm{Paco}}_2; $$

$$ \mathrm{LOI}=\left(\mathrm{Jugular}\kern0.17em \mathrm{venous}\kern0.17em \mathrm{bulb}\kern0.17em \mathrm{lactate}-\mathrm{arterial}\kern0.17em \mathrm{lactate}\right)/{\mathrm{AJDo}}_2. $$

Biomarkers of serum neuron specific enolase (NSE) and central nervous system specific protein (S100β) were measured with enzyme-linked immunosorbent assay kits (Rapid Bio, USA) at baseline, ROSC 1 h, 6 h, and 12 h, respectively.

### Statistical analysis

Continuous variables were shown as mean and standard deviation (SD) or median and interquartile range (IQR). Categorical variables were reported as numbers and percentages. Categorical data were compared with the Chi-square test. Continuous data for normality were checked and compared with the Student’s t-test for the normal distribution and with the Mann-Whitney U test for the non-normal distribution. The EtCO_2_, MAP, ICP, Pbto_2_, Tbt, gradient of Pjvo_2_ and Pbto_2_, difference of Pjvco_2_ and Paco_2_, CO, EVLW, NSE and S100β between hypercapnia and normocapnia group over time were compared using a generalized mixed model with a compound symmetry covariance matrix. We performed all the statistical analyses with R software (version 3.5.1, www.r-project.org). R package “lme4” was used for statistical analyses of generalized mixed model. A two-sided *p* value of less than 0.05 was considered statistically significant.

## Results

**Fig. 2:**
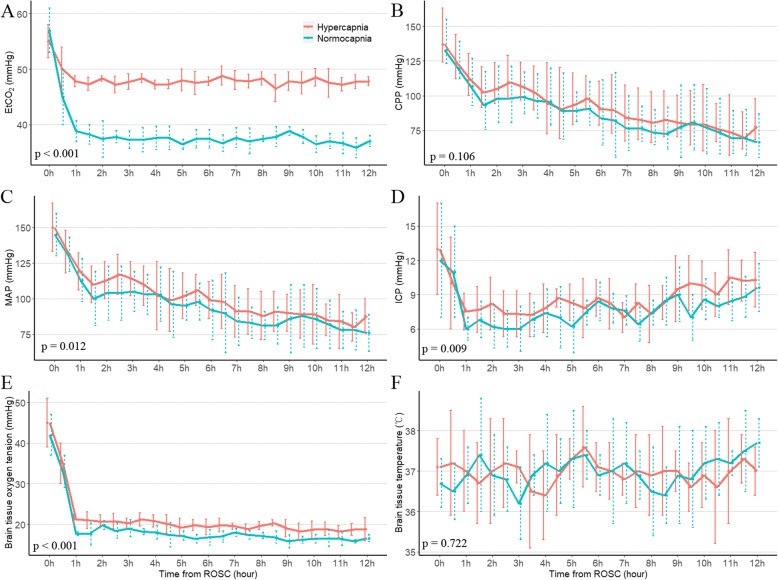
End-tidal carbon dioxide (EtCO_2_), cerebral perfusion pressure (CPP), mean arterial pressure (MAP), intracranial pressure (ICP), brain tissue oxygen tension, and brain tissue temperature during the study period between mild hypercapnia group and hypercapnia group. Data were expressed as mean ± SD (error bars). *P* value were calculated using generalized mixed models

**Fig. 3:**
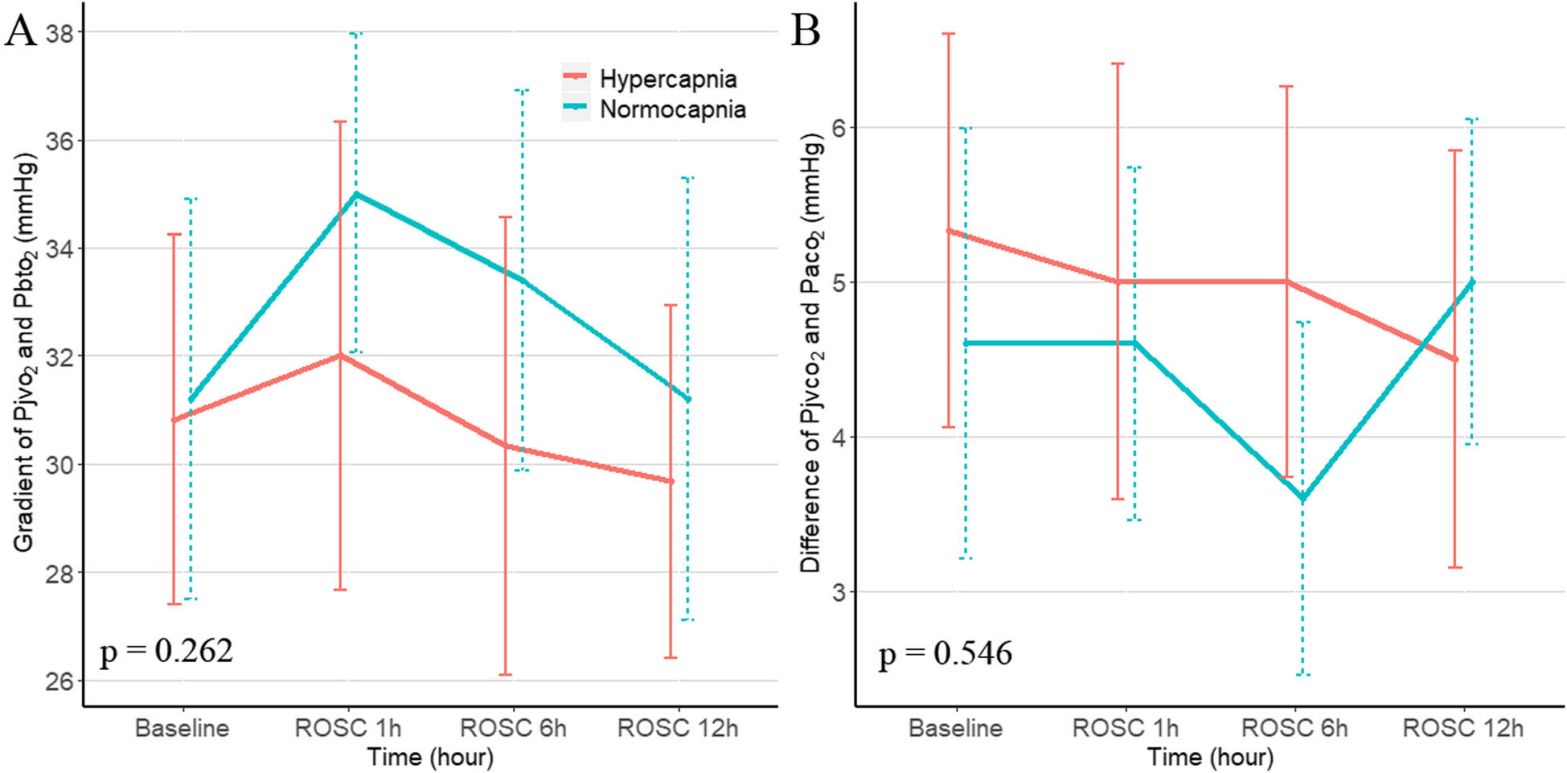
Gradient of jugular venous bulb oxygen tension (Pjvo_2_) and brain tissue oxygen tension (Pbto_2_) and difference of jugular venous partial pressure of carbon dioxide (Pjvco_2_) and arterial partial pressure of carbon dioxide (Paco_2_) at baseline, ROSC 1 h, 6 h, and 12 h post-resuscitation. Data were shown as mean ± SD (error bars). *P* values were calculated using generalized mixed models

**Fig. 4:**
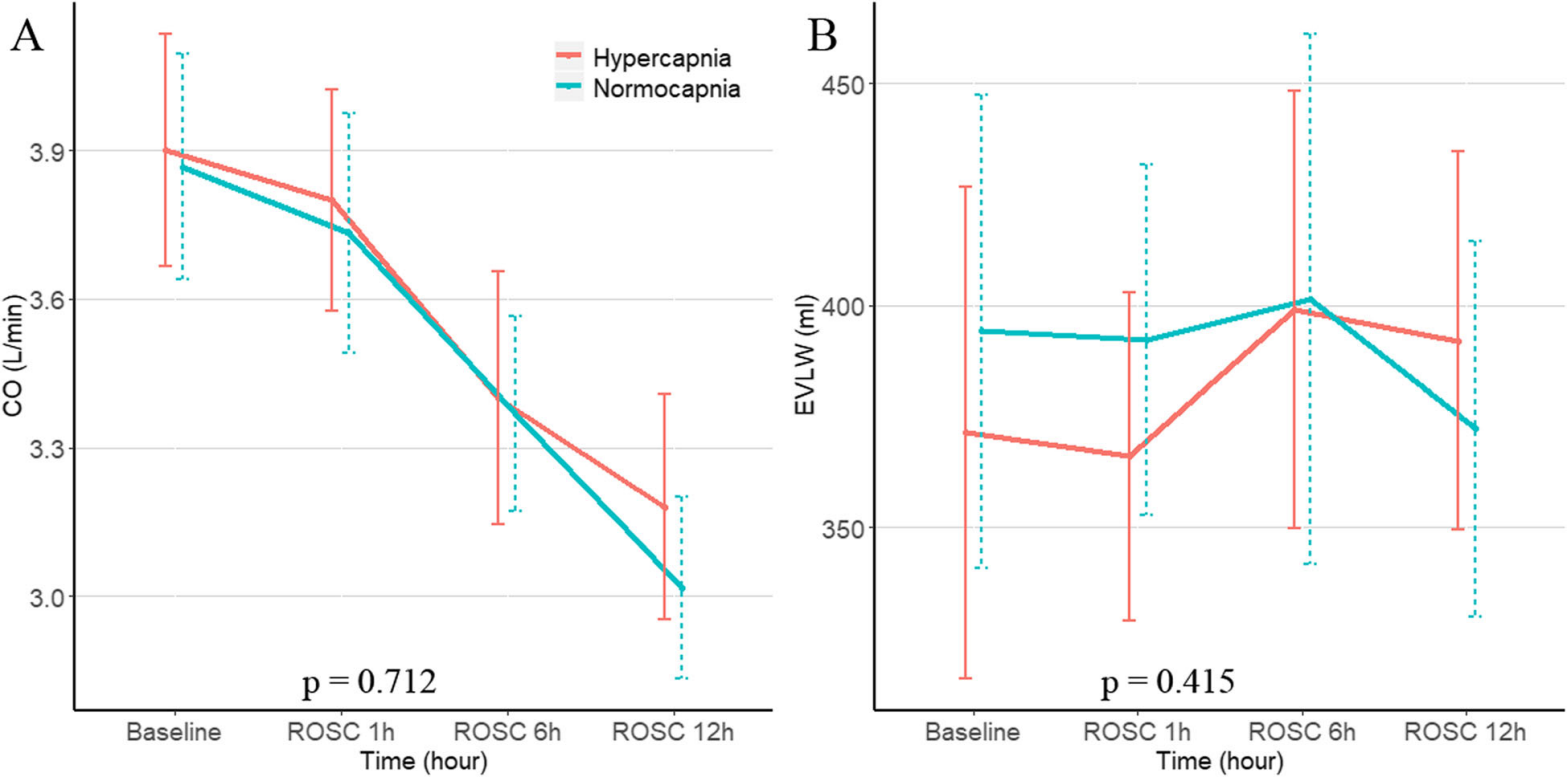
Cardiac output (CO) and extravascular lung water (EVLW) at baseline, ROSC 1 h, 6 h, and 12 h post-resuscitation between hypocapnia and mild hypercapnia group. Data were expressed as mean ± SD (error bars). *P* values were calculated using generalized mixed models

**Fig. 5:**
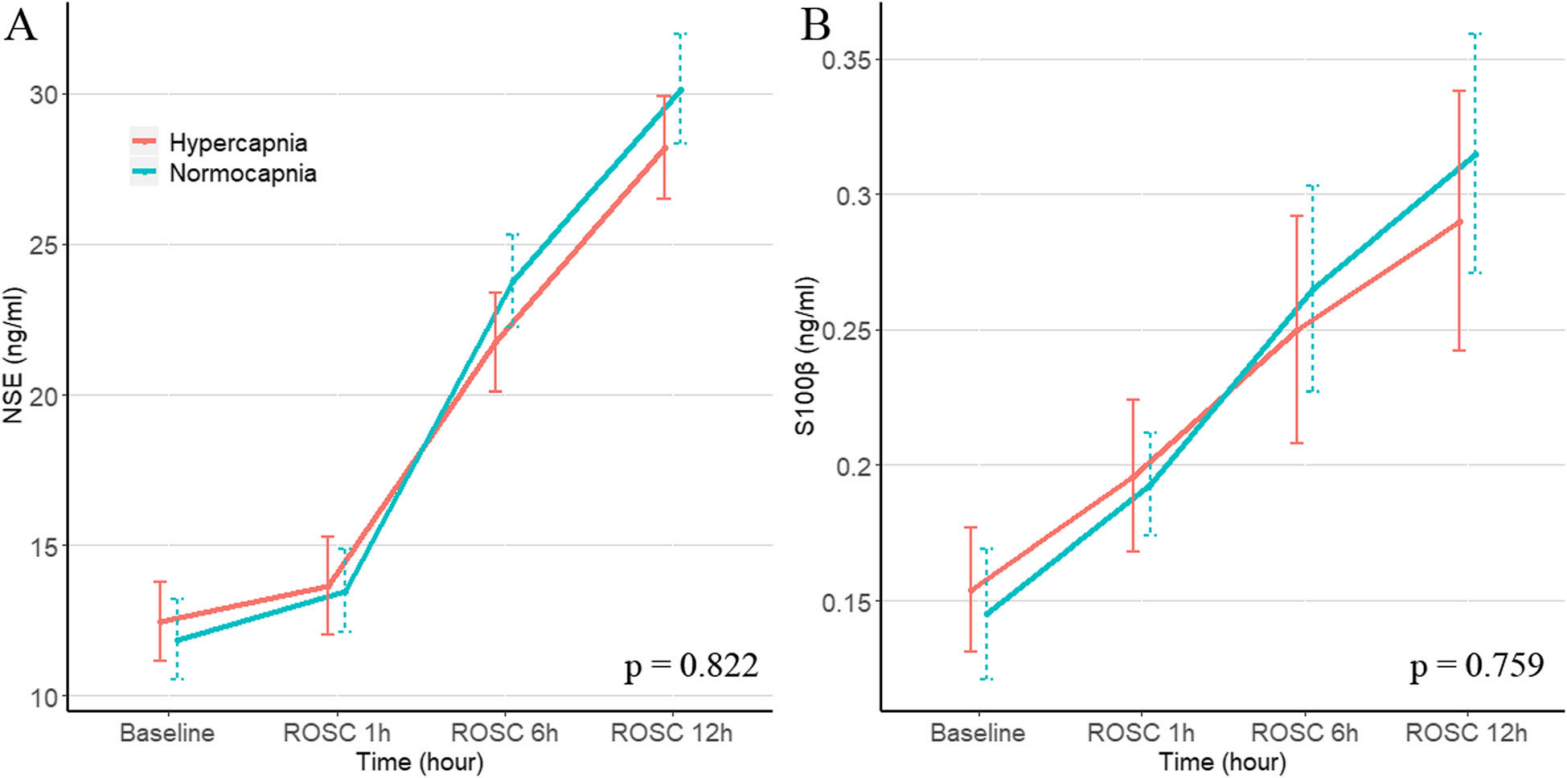
Neuron-specific enolase (NSE) and central nervous system specific protein (S100β) at baseline, ROSC 1 h, 6 h, and 12 h post-resuscitation between hypocapnia group and mild hypercapnia group. Data were shown as mean ± SD (error bars). *P* values were calculated using generalized mixed models

Twelve (75%) of the sixteen pigs were successfully resuscitated and were randomized into the normocapnia (*n* = 6) and mild hypercapnia (*n* = 6) group. One of the pigs in the normocapnia group had recurrent CA after ROSC and was not successfully resuscitated. A total of eleven pigs survived during the study period. No significant differences in body weight, MAP, CO, EVLW, ICP, Pbto_2_, Tbt, hemoglobin, arterial and jugular venous bulb blood gas analyses, NSE, and S100β were observed between the normocapnia and mild hypercapnia groups at baseline (Table 1 and Figs. 2, 3, 4, 5).

**Table 1:** Comparison of characteristics between mild hypercapnia and normocapnia

Variables	Hypercapnia (*n* = 6)	Normocapnia (*n* = 6)	*P* value
Survival for 12 h	6/6	5/6	1
Weight, kg	39.7 ± 1.2	40 ± 0.9	0.835
Duration of asphyxia, minutes	8.1 ± 0.8	8.3 ± 1.1	0.789
Shocks	1 (0, 2)	1 (1, 2)	1
Duration of CPR, minutes	4.4 ± 1.8	5.6 ± 1.2	0.248
Epinephrine, mg	0.8 ± 0.4	0.9 ± 0.6	0.916
Hemoglobin, g/L	149 ± 7	146 ± 8	0.54
Pao_2_, mmHg			
Baseline	82 ± 12	88 ± 13	0.432
ROSC 1 h	99 ± 14	107 ± 7	0.221
ROSC 6 h	83 ± 4	93 ± 8	0.043
ROSC 12 h	77 ± 4	86 ± 5	0.007
Paco_2_, mmHg			
Baseline	39 ± 2	40 ± 2	0.958
ROSC 1 h	52 ± 3	41 ± 2	< 0.001
ROSC 6 h	51 ± 2	40 ± 1	< 0.001
ROSC 12 h	50 ± 3	39 ± 2	< 0.001
AJDo_2_, vol%			
Baseline	6.3 ± 0. 5	6.1 ± 0. 8	0.666
ROSC 1 h	4.7 ± 1.4	5.7 ± 0.6	0.13
ROSC 6 h	3.9 ± 0.5	4 ± 0.9	0.91
ROSC 12 h	4 ± 1.5	4.3 ± 0.6	0.7
LOI, mmol/L/vol%			
Baseline	0.12 ± 0.05	0.11 ± 0.04	0.807
ROSC 1 h	0.27 ± 0.06	0.21 ± 0.05	0.095
ROSC 6 h	0.22 ± 0.13	0.27 ± 0.11	0.578
ROSC 12 h	0.31 ± 0.11	0.3 ± 0.08	0.974

Data were expressed as mean ± SD (standard deviation) or median (interquartile range). Except for the baseline, data of Pao_2_, Paco_2_, AJDo_2_, and LOI after ROSC were calculated from 5 pigs survived for the study period

*CPR* Cardiopulmonary resuscitation, *ROSC* Return of spontaneous circulation, *Pao*_*2*_ Pressure of arterial oxygen, *Paco*_*2*_ Pressure of arterial carbon dioxide, *AJDo*_*2*_ Arteriojugular oxygen content difference, *LOI* Lactate oxygen index

During CPR, no significant differences were observed in the duration of asphyxia, duration of CPR, times of shock, and amount of epinephrine use (Table 1). Immediately after resuscitation, there was a transient surge in EtCO_2_, MAP, ICP, and Pbto_2_ (Fig. 2). However, they gradually stabilized within 1 h.

After resuscitation, there were significant differences in EtCO_2_ and Paco_2_ during the 12 h of treatment. Pigs in the mild hypercapnia group showed a trend toward a lower Pao_2_ (ROSC 6 h and 12 h) (Table 1). Pbto_2_, MAP and ICP were significantly higher in the mild hypercapnia group compared to the normocapnia ventilation group using a generalized mixed model with a compound-symmetry covariance matrix. (Fig. 2 and Supplemental Tables 1, 2, 3). The two groups had similar CPP and Tbt during the study period (Fig. 2). No effect of mild hypercapnia ventilation on CO and EVLW were observed (Fig. 4).

There were no significant differences in gradient of Pjvo_2_ to Pbto_2_ and ΔPco_2_ when comparing normocapnia group with mild hypercapnia group (Fig. 3). As for secondary derivatives of metabolism, no differences were observed in AJDo_2_ and LOI between two groups (Table 1).

There was a significant increase in NSE and S100β over time (*p* <  0.001 for both biomarkers) (Supplemental Table 4). However, no differences were found between the normocapnia group and mild hypercapnia group (Fig. 5).

## Discussion

The present study investigated the effect of mild hypercapnia (EtCO_2_ 45 ~ 50 mmHg) after successful resuscitation, which was obtained by adjusting respiratory rate, on Pbto_2_, diffusion limitation, and cerebral metabolism. The results showed short term mild hypercapnia post-resuscitation could improve Pbto_2_; however, mild hypercapnia was associated with higher ICP and was not associated with improved Pjvo_2_ to Pbto_2_ gradient. In addition, mild hypercapnia was not associated with attenuation of neurological biomarkers of NSE and S100β in this asphyxial cardiac arrest model. These results may challenge the possible beneficial effect of mild hypercapnia on neurological recovery in hypoxic ischemic brain injury postcardiac arrest.

Respiratory care plays an important role for postcardiac arrest patients; however, no specific target range for Paco_2_ has been suggested. The resuscitation guidelines recommend normocapnia (EtCO_2_ 30 ~ 40 mmHg or Paco_2_ 35 ~ 45 mmHg) may be a reasonable goal based on inconsistent results [8]. Recently, several studies have investigated the effect of mild hypercapnia on postcardiac arrest care; however, the results were not completely consistent [9–11, 13, 15]. The “Carbon Control after Cardiac Arrest (CCC) trial” found mild hypercapnia attenuated the release of NSE compared with normocapnia [11], whereas some experimental and randomized pilot trials did not observe the effect of mild hypercapnia on NSE or S100β [9, 10]. Meanwhile, there was one consistent finding that mild hypercapnia increased regional cerebral tissue oxygen saturation (Scto_2_) assessed by near-infrared spectroscopy (NIRS) [9, 13]. The results of the present study were in line with the previous studies. We found no difference in the effect of mild hypercapnia and normocapnia on NSE or S100β; moreover, mild hypercapnia was associated with higher Pbto_2_ which was monitored by invasive measurement.

Continuous brain oxygenation monitoring had been used both during CPR and post-ROSC with non-invasive cerebral oximetry [20, 21]. Higher Scto_2_ levels at initiation of resuscitation and during CPR were associated with resuscitation success and may reflect high-quality CPR [21, 22]. However, Scto_2_ was not found to be predictive of good neurological outcome during the post-arrest period [21]. That partly attributes to Scto_2_ assessed by NIRS may not correlate with invasively measured Pbto_2_ values [16, 23]. To the best of our knowledge, this was the first study to report the Pbto_2_ values between mild hypercapnia and normocapnia ventilation in postcardiac arrest model. Nevertheless, more studies need to be conducted to determine whether a high Pbto_2_ would increase the likelihood of a favorable neurological outcome.

Hypercapnia could augment Pbto_2_ in anesthetized rats, by mechanisms which may include incremental increases in CBF and Pao_2_ [24]. However, in the present study, we found Pao_2_ was higher in normocapnia than mild hypercapnia ventilation at ROSC 6 h and 12 h, which was not in line with Hare’s study [24]. We induced mild hypercapnia by adjusting the respiratory rate, which decreased the minute ventilation. Lower minute ventilation may cause lower Pao_2_. The results of the present study showed mild hypercapnia increased Pbto_2_ but not Pao_2_, which may suggest the effect of mild hypercapnia on Pbto_2_ mainly attribute to increased CBF or other potential mechanisms. Previous studies showed Paco_2_ is the major physiological regulator of CBF and a higher Paco_2_ could increase CBF [12]. In our study, we did not monitor the CBF, and could not comment on whether there was any difference in CBF between mild hypercapnia and normocapnia in asphyxial CA model. Of note, we found mild hypercapnia had higher MAP, which may be associated with increased CBF.

The increased gradient of Pjvo_2_ and Pbto_2_ had been suggested to be associated with diffusion limitation [25, 26]. In the present study, we did not find the difference of gradient of Pjvo_2_ and Pbto_2_ between mild hypercapnia and normocapnia. Besides, there were no differences in AJDo_2_, ΔPco_2_, and LOI between mild hypercapnia and normocapnia groups. AJDo_2_ and ΔPco_2_ had been considered as good indicators of ischemia and hypoperfusion [27–29]. LOI levels were also reported to be associated with CBF and related to the outcome of head-injured patients [30, 31]. The findings may suggest that mild hypercapnia does not improve tissue perfusion or diffusion limitation, which could explain the no differences of biomarkers of NSE or S100β between two groups. However, due to the absence of pathologic tissue test or gold standard measures of cerebral metabolism, we were unable to further explore the potential mechanisms.

The side effect of hypercapnia should also need to be mentioned. Hypercapnia increases ICP, with hypocapnia used as a temporizing measure to ameliorate intracranial hypertension [32]. In our study, there was a slightly increased MAP and ICP in mild hypercapnia group. The result of a higher MAP in the mild hypercapnic animals was consistent with previous studies [10, 33]. However, there were studies suggesting no association between hypercapnia and MAP [13, 34]. Hypercapnia may have effects on cardiovascular system, like inhibiting cardiac and vascular muscle contractility; however, the effects could be counterbalanced by an increase in the sympathetic tone [12, 35]. Although the slightly increased ICP in the mild hypercapnia group was expected, CPP was not significantly different between the two groups. In addition, there were no differences in CO and EVLW between the two groups.

There are several limitations to this study. First, we used asphyxial CA model instead of CA of VF model, which was widely used for establishing CA model. Previous studies showed asphyxial CA model causes more severe cerebral metabolism injuries and ischemic brain injury after asphyxial CA appears more severe and more widespread than VF model of the same duration [36, 37]. Second, we defined mild hypercapnia as EtCO_2_ between 45 and 50 mmHg based on previously published studies [10], which may be arbitrary, since the target EtCO_2_ levels associated with potential benefit or harm are unknown. Third, we only assessed two different levels of EtCO_2_ for a period of 12 h. Fourth, Pbto_2_ is a focal measure, which could not represent the global monitor. The pathophysiology of CA is global cerebral ischemia. Fifth, the observational period was only 12 h in our study. The effect of mild hypercapnia on long-term outcomes may need further investigation. Sixth, the lack of pathology and cerebral blood flow data limited the interpretation of the potential mechanisms. Finally, we did not implement targeted temperature management (TTM) after ROSC, which is strongly recommended by resuscitation guidelines. That may merit further research.

## Conclusions

Short term mild hypercapnia post-resuscitation could improve brain tissue oxygen tension. However, no corresponding improvements in the gradient of jugular venous bulb oxygen tension to brain tissue oxygen tension and biomarkers of neurological recovery were observed in the porcine asphyxial cardiac arrest model.

## Supplementary information

**Additional file 1: Table S1.** Comparisons of Pbto_2_ at different time points between mild hypercapnia and normocapnia. **Table S2.** Comparisons of MAP at different time points between mild hypercapnia and normocapnia. **Table S3.** Comparisons of ICP at different time points between mild hypercapnia and normocapnia. **Table S4.** Comparisons of NSE and S100β between mild hypercapnia and normocapnia.

## Data Availability

The datasets during the present study are available from the corresponding author on reasonable request.

## Abbreviations

ABG: Arterial blood gas
AJDo_2_: Arteriojugular oxygen difference
CA: Cardiac arrest
CBF: Cerebral blood flow
CO: Cardiac output
CPP: Cerebral perfusion pressure
CPR: Cardiopulmonary resuscitation
CVP: Central venous pressure
EtCO_2_: End-tidal carbon dioxide
EVLW: Extravascular lung water
ICP: Intracranial pressure
IQR: Median and interquartile range
LOI: Lactate oxygen index
MAP: Mean arterial blood pressure
NIRS: Near infrared spectroscopy
NSE: Neuron specific enolase
Paco_2_: Partial pressure of arterial carbon dioxide
Pao_2_: Pressure of arterial oxygen
Pbto_2_: Brain tissue oxygen tension
PCAS: Post-cardiac arrest syndrome
Pjvco_2_: Pressure of jugular venous bulb carbon dioxide
Pjvo_2_: Partial pressure of jugular venous bulb oxygen
ROSC: Return of spontaneous circulation
Sao_2_: Saturation of arterial oxygen
Scto_2_: Regional cerebral tissue oxygen saturation
SD: Standard deviation
Sjvo_2_: Saturation of jugular venous bulb oxygen
Tbt: Brain tissue temperature
VF: Ventricular fibrillation
ΔPco_2_: Difference of Pjvco_2_ to Paco_2_
