# Supplementary material for: Mild hypercapnia improves brain tissue oxygen tension but not diffusion limitation in asphyxial cardiac arrest: an experimental study in pigs
Source: BMC Anesthesiol. 2020 Sep 29;20:252. doi: 10.1186/s12871-020-01162-z (PMC7522908; doi:10.1186/s12871-020-01162-z)
Supplement: Supplementary file 1 — Additional file 1: Table S1. Comparisons of Pbto2 at different time points between mild hypercapnia and normocapnia. Table S2. Comparisons of MAP at different time points between mild hypercapnia and normocapnia. Table S3. Comparisons of ICP at different time points between mild hypercapnia and normocapnia. Table S4. Comparisons of NSE and S100β between mild hypercapnia and normocapnia. [file 12871_2020_1162_MOESM1_ESM.docx]

**Mild hypercapnia improves brain tissue oxygen tension but not diffusion limitation in asphyxial cardiac arrest: An experimental study in pigs**

Dawei Zhou, Zhimin Li, Shaolan Zhang, Lei Wu, Yiyuan Li,

Guangzhi Shi, Jianxin Zhou

**Supplementary materials**

Supplemental Table 1 Comparisons of Pbto_2_ at different time points between mild hypercapnia and normocapnia

| Pbto_2_, mmHg | Hypercapnia (n = 6) | Normocapnia (n = 5) | P value |
| --- | --- | --- | --- |
| ROSC | 45 ± 6.2 | 42 ± 5.1 | 0.398 |
| ROSC 30 min | 35 ± 5.2 | 33 ± 4.2 | 0.489 |
| ROSC 60 min | 21.2 ± 1.3 | 17.6 ± 0.5 | < 0.001 |
| ROSC 90 min | 21 ± 2 | 17.6 ± 2.7 | 0.040 |
| ROSC 120 min | 20.7 ± 1.6 | 19.8 ± 1.1 | 0.316 |
| ROSC 150 min | 20.8 ± 1.9 | 18.4 ± 1.9 | 0.067 |
| ROSC 180 min | 20.3 ± 1.2 | 19 ± 2.1 | 0.228 |
| ROSC 210 min | 21.2 ± 1.6 | 18.2 ± 0.8 | 0.004 |
| ROSC 240 min | 20.8 ± 1.5 | 18 ± 1.7 | 0.017 |
| ROSC 270 min | 20 ± 2.3 | 17.4 ± 3 | 0.138 |
| ROSC 300 min | 19.2 ± 2.3 | 17.2 ± 1.1 | 0.110 |
| ROSC 330 min | 19.8 ± 1.3 | 16.4 ± 1.1 | 0.001 |
| ROSC 360 min | 19.3 ± 1.9 | 16.8 ± 1.9 | 0.058 |
| ROSC 390 min | 19.8 ± 1.7 | 17 ± 1.4 | 0.017 |
| ROSC 420 min | 19.5 ± 0.8 | 18 ± 1.2 | 0.022 |
| ROSC 450 min | 18.8 ± 1.2 | 17.4 ± 1.1 | 0.077 |
| ROSC 480 min | 19.7 ± 1 | 17.2 ± 0.8 | 0.001 |
| ROSC 510 min | 20.3 ± 0.8 | 16.8 ± 1.5 | 0.001 |
| ROSC 540 min | 19 ± 2.1 | 15.8 ± 1.6 | 0.021 |
| ROSC 570 min | 18.2 ± 2.1 | 16.2 ± 0.8 | 0.077 |
| ROSC 600 min | 18.7 ± 2.1 | 16.4 ± 1.1 | 0.056 |
| ROSC 630 min | 18.8 ± 1.7 | 16.6 ± 2.1 | 0.086 |
| ROSC 660 min | 18.2 ± 1.5 | 16.4 ± 1.7 | 0.095 |
| ROSC 690 min | 18.7 ± 1.4 | 15.8 ± 0.4 | 0.002 |
| ROSC 720 min | 18.8 ± 2.8 | 16.6 ± 0.9 | 0.129 |

Data were expressed as mean ± SD (standard deviation).

Pbto_2_ brain tissue oxygen tension, ROSC return of spontaneous circulation.

Supplemental Table 2 Comparisons of MAP at different time points between mild hypercapnia and normocapnia

| MAP, mmHg | Hypercapnia (n = 6) | Normocapnia (n = 5) | P value |
| --- | --- | --- | --- |
| ROSC | 149 ± 17 | 140 ± 15 | 0.381 |
| ROSC 30 min | 132 ± 15 | 131 ± 12 | 0.907 |
| ROSC 60 min | 119 ± 12.5 | 107 ± 8.6 | 0.045 |
| ROSC 90 min | 110 ± 9.2 | 101 ± 18.7 | 0.323 |
| ROSC 120 min | 113 ± 13.1 | 104 ± 18.5 | 0.369 |
| ROSC 150 min | 116 ± 13.6 | 105 ± 19.3 | 0.296 |
| ROSC 180 min | 112 ± 11.7 | 106 ± 13.7 | 0.453 |
| ROSC 210 min | 110 ± 13.7 | 103 ± 13.7 | 0.421 |
| ROSC 240 min | 103 ± 24 | 102 ± 19.2 | 0.942 |
| ROSC 270 min | 99 ± 21.7 | 96 ± 22.5 | 0.827 |
| ROSC 300 min | 102 ± 15.9 | 95 ± 10.2 | 0.419 |
| ROSC 330 min | 105 ± 10.5 | 96 ± 12.9 | 0.233 |
| ROSC 360 min | 99 ± 5.8 | 85 ± 10.9 | 0.055 |
| ROSC 390 min | 98 ± 18.5 | 83 ± 28.2 | 0.316 |
| ROSC 420 min | 88 ± 18.2 | 82 ± 6.9 | 0.507 |
| ROSC 450 min | 87 ± 15.9 | 81 ± 11 | 0.495 |
| ROSC 480 min | 83 ± 17.3 | 80 ± 10 | 0.741 |
| ROSC 510 min | 86 ± 14.1 | 79 ± 13.3 | 0.422 |
| ROSC 540 min | 85 ± 12.6 | 83 ± 24.3 | 0.864 |
| ROSC 570 min | 85 ± 16.9 | 84 ± 21.5 | 0.933 |
| ROSC 600 min | 84 ± 8.7 | 83 ± 20.9 | 0.917 |
| ROSC 630 min | 83 ± 11.3 | 82 ± 24.2 | 0.930 |
| ROSC 660 min | 82 ± 18.5 | 78 ± 13.1 | 0.695 |
| ROSC 690 min | 80 ± 10.4 | 79 ± 16.5 | 0.905 |
| ROSC 720 min | 83 ± 11.6 | 76 ± 12.4 | 0.211 |

Data were expressed as mean ± SD (standard deviation).

MAP mean arterial pressure, ROSC return of spontaneous circulation.

Supplemental Table 3 Comparisons of ICP at different time points between mild hypercapnia and normocapnia

| ICP, mmHg | Hypercapnia (n = 6) | Normocapnia (n = 5) | P value |
| --- | --- | --- | --- |
| ROSC | 13 ± 4 | 12 ± 5 | 0.720 |
| ROSC 30 min | 10 ± 4 | 11 ± 4 | 0.689 |
| ROSC 60 min | 7.8 ± 1.2 | 6 ± 1 | 0.043 |
| ROSC 90 min | 7.7 ± 2 | 6.8 ± 1.6 | 0.438 |
| ROSC 120 min | 8.2 ± 2.3 | 6.2 ± 1.9 | 0.156 |
| ROSC 150 min | 7.3 ± 2 | 6 ± 1.6 | 0.272 |
| ROSC 180 min | 7.3 ± 2 | 6 ± 2 | 0.311 |
| ROSC 210 min | 7.2 ± 1.9 | 6.8 ± 1.5 | 0.712 |
| ROSC 240 min | 7.8 ± 2.1 | 7.4 ± 2.1 | 0.760 |
| ROSC 270 min | 8.7 ± 0.8 | 7 ± 2.7 | 0.173 |
| ROSC 300 min | 8.3 ± 1.6 | 6.1 ± 2.3 | 0.038 |
| ROSC 330 min | 7.8 ± 2.6 | 7.4 ± 1.1 | 0.757 |
| ROSC 360 min | 8.7 ± 1.6 | 8.4 ± 1.7 | 0.770 |
| ROSC 390 min | 8.2 ± 2.2 | 7.8 ± 1.3 | 0.730 |
| ROSC 420 min | 7 ± 1.3 | 7.6 ± 1.3 | 0.465 |
| ROSC 450 min | 8.3 ± 1.6 | 6.4 ± 1.5 | 0.075 |
| ROSC 480 min | 7.3 ± 2.5 | 7.4 ± 1.1 | 0.936 |
| ROSC 510 min | 8.3 ± 1.5 | 8.4 ± 2.1 | 0.929 |
| ROSC 540 min | 9.5 ± 2.9 | 9 ± 2.4 | 0.766 |
| ROSC 570 min | 10 ± 2.4 | 7 ± 2 | 0.049 |
| ROSC 600 min | 9.8 ± 2.1 | 8.6 ± 1.8 | 0.341 |
| ROSC 630 min | 9 ± 1.9 | 8 ± 1 | 0.319 |
| ROSC 660 min | 10.5 ± 2.4 | 8.1 ± 1.7 | 0.056 |
| ROSC 690 min | 10.2 ± 1.8 | 8.8 ± 1.8 | 0.231 |
| ROSC 720 min | 10.3 ± 2.4 | 9.6 ± 2.1 | 0.623 |

Data were expressed as mean ± SD (standard deviation).

ICP intracranial pressure, ROSC return of spontaneous circulation.

| Variables | Hypercapnia (n = 6) | Normocapnia (n = 6) | P value |
| --- | --- | --- | --- |
| NSE, ng/ml |  |  |  |
| Baseline | 12.5 ± 1.3 | 11.9 ± 1.3 | 0.465 |
| ROSC 1hour | 13.6 ± 1.6 | 13.5 ± 1.4 | 0.916 |
| ROSC 6hour | 21.7 ± 1.6 | 23.8 ± 1.5 | 0.053 |
| ROSC 12hour | 28.2 ± 1.7 | 30.2 ± 1.8 | 0.091 |
| S100β, ng/ml |  |  |  |
| Baseline | 0.154 ± 0.023 | 0.145 ± 0.024 | 0.542 |
| ROSC 1hour | 0.196 ± 0.028 | 0.193 ± 0.019 | 0.844 |
| ROSC 6hour | 0.25 ± 0.042 | 0.265 ± 0.038 | 0.554 |
| ROSC 12hour | 0.291 ± 0.048 | 0.315 ± 0.044 | 0.395 |

Supplemental Table 4 Comparisons of NSE and S100β between mild hypercapnia and normocapnia

Data were expressed as mean ± SD (standard deviation). Except for the baseline, data of NSE and S100β after ROSC were calculated from 5 pigs survived for the study period.

NSE neuron-specific enolase, ROSC return of spontaneous circulation.
